# WordCloud: a Cytoscape plugin to create a visual semantic summary of networks

**DOI:** 10.1186/1751-0473-6-7

**Published:** 2011-04-07

**Authors:** Layla Oesper, Daniele Merico, Ruth Isserlin, Gary D Bader

**Affiliations:** 1 Department of Computer Science, Brown University, Providence, RI, USA; 2 The Donnelly Centre, University of Toronto, Toronto, ON, Canada; 3 Banting and Best Department of Medical Research, University of Toronto, Toronto, ON, Canada

## Abstract

**Background:**

When biological networks are studied, it is common to look for clusters, i.e. sets of nodes that are highly inter-connected. To understand the biological meaning of a cluster, the user usually has to sift through many textual annotations that are associated with biological entities.

**Findings:**

The WordCloud Cytoscape plugin generates a visual summary of these annotations by displaying them as a tag cloud, where more frequent words are displayed using a larger font size. Word co-occurrence in a phrase can be visualized by arranging words in clusters or as a network.

**Conclusions:**

WordCloud provides a concise visual summary of annotations which is helpful for network analysis and interpretation. WordCloud is freely available at http://baderlab.org/Software/WordCloudPlugin

## Findings

### Introduction

**Figure 1:**
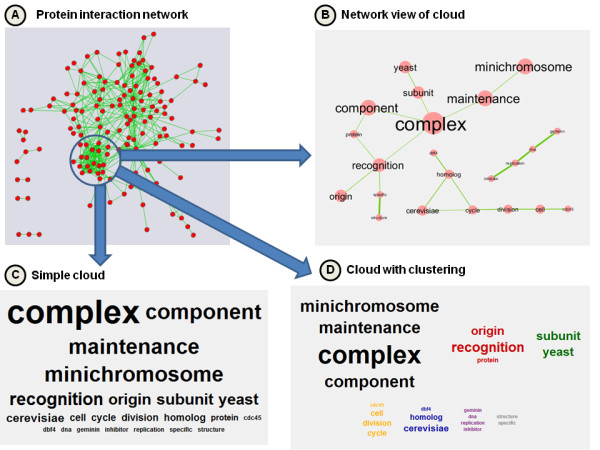
**Tag cloud for a protein interaction cluster**. The network consists of physical interactions between S. cerevisiae proteins involved in DNA replication (A). A group of highly inter-connected proteins was selected (blue circle) and their full names were mined using WordCloud. The results are shown for the three layouts: network (B), simple (C) and clustered (D). "Origin recognition complex component" and "Minichromosome maintenance complex component" are the dominating themes. The corresponding words are ranked on top in the simple cloud layout, but only the clustered and network layout reconstruct the correct connections between them, based on word co-occurrence patterns. Since clustering is non-overlapping, the words "complex" and "component" are forced to appear only in one cluster (with "minichrosome maintenance"), whereas the network layout displays association to "origin recognition" as well.

Networks are widely used to represent relationships between biological entities, such as proteins and genes. Biological networks are typically explored using tools such as Cytoscape [1]. One common analysis consists of identifying sub-networks characterized by a specific feature, such as the presence of dense interconnections compared to the rest of the network [2]. For example, comprehensive maps of protein-protein physical interactions have been mined for dense regions, which represent protein complexes, using clustering algorithms [3]. Once sub-networks have been identified, however, it is often difficult to interpret their biological meaning. Bio-entities typically have rich textual information associated with them, such as Gene Ontology (GO) annotations [4]. A popular method for interpreting sub-networks using this information is enrichment analysis, where node and edge attributes are mined for statistically enriched text terms. For example, a sub-network can be searched for enriched biological pathways associated with the list of nodes. While highly useful, enrichment analysis takes time to perform and produces a simple table of enriched attributes. When deciding which sub-networks are interesting, it is useful to have quick visual feedback displaying frequent node annotation. In previous work, we manually created 'word clouds' to help us with this task [5]. The purpose of the WordCloud plugin is to automatically generate concise visual summaries of such textual attributes for fast access during network exploration (Figure 1).

The WordCloud plugin implements a visual information retrieval system known as a tag cloud. Tag cloud systems are used in a variety of domains from social bookmarking services [6] to summarization of PubMed database searches [7]. The WordCloud implementation extends the basic tag cloud concept of a simple collection of words by also displaying information about word co-occurrence [8,9].

**Figure 2:**
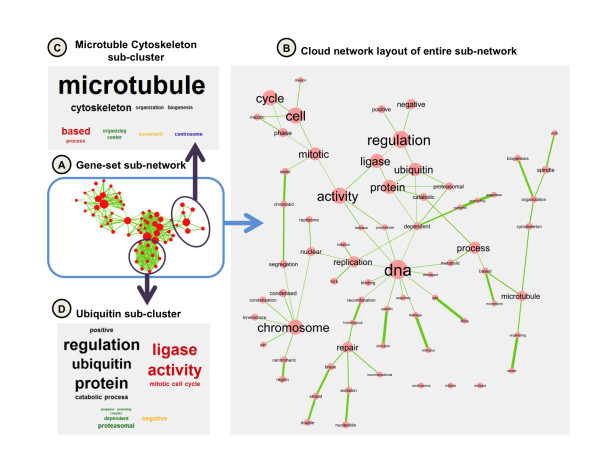
**Application of WordCloud to gene-set enrichment analysis results**. The transcriptional response of breast cancer cells to estrogen treatment was analyzed for gene-set enrichment, as described in [11]. Gene-sets were then arranged as a network using the Enrichment Map visualization technique [11]; edges represent gene-set overlap and clusters correspond to functional groups. A sub-network (A) was selected and analyzed using the WordCloud network layout (B). The most frequent words in gene-set names are "Mitotic Cell Cycle", "DNA Replication", "Ubiquitin Ligase Activity/Regulation", "Chromosome", "Microtubule"; this suggests that the sub-network consists of gene-sets involved in the control of cell proliferation. Specific parts of the sub-network (purple circles) relate to specific functional groups, as suggested by clustered word clouds (C,D).

WordCloud can also be used in combination with enrichment analysis to summarize any type of gene list. Gene-set enrichment analysis is a popular approach to functionally characterize gene lists [10], including gene clusters from protein networks. Known gene-sets, typically derived from standardized annotation systems such as the Gene Ontology, are statistically tested for overrepresentation in the query gene list. However, enrichment analysis can often produce long lists of enriched gene-sets, which are often redundant or interrelated, thus hindering the interpretation of the results. To overcome this problem, several visualization methods have been developed to arrange gene-sets as similarity networks, where clusters correspond to functionally related gene-sets [11-13]. WordCloud can be effectively used to summarize these gene-set clusters (Figure 2).

### Methods and Implementation

WordCloud is a freely available, open source Cytoscape plugin written in Java and compatible with Cytoscape versions 2.6, 2.7 and 2.8. Given a user-defined node selection (i.e. a sub-network), a word cloud can be generated using one or more user-selected node attributes that are of type *string *or *list of string*. Input text from all selected attributes is collected and broken down into words using separation characters, such as punctuation and space delimiters. Flagged words, such as commonly occurring English words and numbers, can be removed. In addition, words that share the same stem (e.g. cell and cells) can be mapped to that stem using the Porter Stemming Algorithm [14]. Font size for all words is then calculated proportionally to word frequency in the input text. The user can optionally scale font size using 'network-weighting' which considers word frequencies of all text in the entire network, rather than just the node selection, to penalize words that appear frequently outside the node selection. In this case, the font size of any word *w *in a tag cloud is directly proportional to:

where *sel*_*w *_is the number of selected nodes that contain the word *w*, *sel*_*tot *_is the total number of selected nodes, *net*_*w *_is the number of nodes in the entire network that contain the word *w*, *net*_*tot *_is the total number of nodes in the network, and *k *is the network normalization coefficient, which can be tuned by the user through an interactive slider bar.

The WordCloud plugin supports several layout options for the tag cloud. The most basic layout consists of the sequence of words arranged in order of descending frequency. The clustered and network layouts offer semantically richer summaries by considering co-occurrence patterns between words. Clusters are built by step-wise aggregation of frequently co-occurring word pairs. Specifically, the WordCloud plugin uses a greedy clustering algorithm similar to hierarchical clustering. Every ordered pair of words {*w*_*1*_*, w*_*2*_} that appear next to each other in at least one of the selected nodes is assigned a similarity score, defined by the ratio of the observed joint probability of these words appearing next to each other in the specified order, to the expected independent probability of these words appearing next to each other:

Each word starts in its own cluster. Next, the most similar word pair is merged to form a larger cluster, maintaining word order, and the process is repeated. Similarity between multi-word clusters is defined as the similarity of the last word appearing in the first cluster and the first word appearing in the second cluster. This helps maintain the order of words in the cluster in the standard left to right English text direction. The cluster merging process is bounded by a user-defined threshold on the word pair similarity score.

Cluster order is determined by the number of words in a cluster and word frequency information. For any word *w *appearing in a tag cloud, *s*(*w*) is the font size assigned to word *w*. A clustered tag cloud consists of a set of clusters *C *= {*C*_*1*_, ..., *C*_*m*_} where each *C*_*i *_contains some set of words {*w*^*i*^_*1*_, ..., *w*^*i*^_*n*_}. The clusters are laid out in decreasing order according to the following value:

This is the *L2 *norm (i.e. Euclidean length) of the cluster's word size vector.

The greedy clustering algorithm described above does not consider the co-occurrence of all word pairs in the input text. Thus, as an alternative to the clustered layout, words can be visualized as a similarity network. Each word is represented as a node, with node and label size proportional to word frequency as previously described. Words are connected by edges whose width is proportional to their similarity score, as defined above. The resulting network can be laid out, analyzed and clustered using Cytoscape functionalities. The network layout is particularly useful when words tend to have multiple co-occurrence partners, rather than a single one.

## Conclusions

WordCloud is a configurable tool for creating quick visual summaries of sub-networks within Cytoscape and is a useful tool to aid interactive network exploration. The configuration options provide a high degree of control over tag cloud visualization resulting in a publication quality summary of a sub-network. WordCloud also includes clustered tag cloud and word similarity network visualization options that retain the meaning of phrases by maintaining word order, rather than just displaying individual words.

## Availability and Requirements

Project name: WordCloud

Project home page: http://baderlab.org/Software/WordCloudPlugin

Operating system: Platform independent

Programming language: Java

Other requirements: Cytoscape version 2.6 or newer, Java SE 5

License: GNU LGPL

Any restrictions to use by non-academics: None

## Competing interests

The authors declare that they have no competing interests.

## Authors' contributions

LO designed and developed the software and drafted the manuscript. DM, RI and GDB conceived the project, contributed to the design of the software and aided in the drafting of the manuscript. All authors have read and approved the final manuscript.
